# Association of Combined PFOA, PFOS, Metals and Allostatic Load on Hepatic Disease Risk

**DOI:** 10.3390/jox14020031

**Published:** 2024-04-28

**Authors:** Mary Balogun, Emmanuel Obeng-Gyasi

**Affiliations:** 1 Department of Built Environment, North Carolina A&T State University, Greensboro, NC 27411, USA; 2 Environmental Health and Disease Laboratory, North Carolina A&T State University, Greensboro, NC 27411, USA

**Keywords:** PFAS, metals, allostatic load, stress, PFOA, PFOS, lead, cadmium, mercury

## Abstract

This study utilizes the National Health and Nutrition Examination Survey (NHANES) 2017–2018 data to explore the relationship between exposure to perfluoroalkyl substances (specifically perfluorooctanoic acid (PFOA) and perfluorooctanesulfonic acid (PFOS), metals lead (Pb), mercury (Hg), and cadmium (Cd), allostatic load, and hepatic disease markers, including the fatty liver index a measure of the likelihood of non-alcoholic fatty liver disease, aspartate aminotransferase (AST), alanine aminotransferase (ALT), alkaline phosphatase (ALP), and total bilirubin. The paper identified significant associations and interaction effects by employing descriptive statistics, Spearman’s correlation analysis, linear regression, and Bayesian kernel machine regression (BKMR). Descriptive statistics highlight sex-specific differences in contaminant levels. Spearman’s analysis underscores strong correlations among metals and per- and polyfluoroalkyl substances (PFAS). Linear regression reveals significant impacts of specific contaminants on AST, ALT, ALP, and bilirubin levels, adjusting for age and alcohol consumption. BKMR results further elucidate the complex, potentially synergistic relationships between these environmental exposures and the likelihood of non-alcoholic fatty liver disease, offering nuanced insights into their combined effects on liver health. The findings emphasize the intricate dynamics of environmental exposures on hepatic function, advocating for targeted public health interventions.

## 1. Introduction

### 1.1. Context

#### 1.1.1. Hepatic Disease: An Issue of Significant Public Health Concern

Hepatic disease has emerged as a fast-growing global health concern with many implications [1,2]. The prevalence of hepatic diseases has been on an alarming rise over the years, ranging from geographical boundaries and affecting diverse human populations [1,3]. This worrisome trend has not only placed a significant burden on healthcare systems globally but has also cast a shadow on the overall well-being and productivity of individuals and communities across the globe [1,4]. It is important to note that hepatic disease has a global influence that reaches beyond the healthcare sphere. These diseases represent significant economic and social issues, such as increased healthcare costs, decreased work productivity, chances of mortality, and a lower quality of life for affected persons and their families [1,2,5]. Furthermore, the burden of hepatic illnesses does not stop with the patients; it spreads throughout society, affecting careers, employers, and healthcare providers. 

Scholarly work by Dreher and colleagues [6] found that hepatic diseases are closely intertwined with various factors like changes in dietary patterns, sedentary lifestyles, the obesity epidemic, and an increase in alcohol consumption. Furthermore, there is growing concern about the influence of environmental toxins and chemicals on liver health. Within this context, the role of perfluorooctanoic acid (PFOA) and perfluorooctane sulfonic acid (PFOS) has come under scrutiny due to their widespread presence and potential health risks [5]. This growing problem makes it critical to identify the significant factors contributing to hepatic disorders and their impact.

#### 1.1.2. PFAS and Metals Exposure—Environmental Chemicals of Concern

##### PFAS Exposure

Per- and polyfluorinated substances (PFAS), such as PFOA and PFOS, are pervasive environmental contaminants found in numerous everyday items like paint, toys, nonstick cookware, and firefighting foams [5,7,8]. Due to their chemical stability, PFAS persists in the environment, contaminating water sources and wildlife and accumulating within human bodies [9,10]. This persistence poses significant health concerns as PFAS can accumulate in human tissues over time, particularly in the liver [4,8,11,12]. Exposure to PFAS occurs through various routes, including ingestion of contaminated food and water, inhalation of airborne particles, or direct contact with PFAS-containing products [4,8,13,14].

##### Metals Exposure

In addition to PFAS, metals such as lead (Pb), mercury (Hg), and cadmium (Cd) also pose significant environmental and health risks. These metals are prevalent in various products and industries and can contaminate the environment, including water bodies and soil [5,7,8]. Like PFAS, metals persist in the environment and can accumulate in ecosystems and within human tissues over time [11,12]. Metals exposure occurs through pathways similar to PFAS, including ingestion, inhalation, and direct contact with contaminated materials [13,14].

##### Co-Exposure of PFAS and Metals

The simultaneous presence of PFAS and metals in the environment exacerbates the risks associated with individual exposures. Their widespread use and persistence result in continuous release into air, water, and soil, posing a significant public health concern [14,15,16,17]. Co-exposure to PFAS and metals can lead to additive or synergistic effects, potentially magnifying adverse health outcomes [7]. Therefore, understanding the separate impacts of PFAS and metals exposure before addressing their co-exposure is essential for elucidating their combined effects on human health and the environment.

PFAS and metal’s widespread use and environmental persistence speak to their effect on populations over their life course [8]. Specifically, the persistence allows PFAS and metals to accumulate in ecosystems, including water bodies, soil, and even wildlife [9,10], exposing individuals and communities to various doses and combinations of mixtures. 

#### 1.1.3. Relevance of PFAS and Metals to Hepatic Disease

The relevance of PFAS (per- and polyfluoroalkyl substances) and metals to hepatic disease is becoming increasingly significant in public health due to their rising prevalence and impact on liver health [11,12]. These substances, known for their persistence in the environment and potential for bioaccumulation, are implicated in various liver diseases, including non-alcoholic fatty liver disease and hepatocellular carcinoma. Studies have identified a correlation between exposure to these toxic chemicals and liver dysfunction [11,12].

Globally, liver disease, encompassing conditions like hepatitis, cirrhosis, and liver cancer, is a leading cause of morbidity and mortality. The burden of liver disease is growing, especially in regions with high industrial activity and environmental pollution. For instance, in China, liver cancer is a significant cause of cancer-related deaths linked to industrial pollution [11].

Heavy metals like cadmium (Cd), lead (Pb), and mercury (Hg) are known to disrupt liver function [13]. These metals, prevalent in industrial wastes and atmospheric pollution, can enter the human body through food chains and accumulate in organs, leading to chronic poisoning [14]. Epidemiological studies and animal experiments have consistently demonstrated the harmful impact of heavy metal exposure on liver health, including significant associations with liver damage markers like ALT and AST [15,16,17].

Most existing studies focus on the impact of individual metals on liver health. However, the reality of environmental exposure is often a mixture of several toxic metals. This complex exposure scenario presents a challenge for understanding the combined effects on liver health. A study in rural China addressed this by using Bayesian kernel machine regression to analyze the combined effects of multiple metals (Cr, Co, Cd, Pb) on liver function [11]. This approach revealed that co-exposure to these metals has a measurable impact on liver health, underscoring the need for public health strategies to mitigate the risks of heavy metal pollution.

#### 1.1.4. Individual vs. Combined Exposure

Previous research has examined the health impacts of specific PFAS and metals such as PFOA, PFOS, Hg, Pb, and Cd individually on hepatic outcomes [15,18,19,20,21]. However, these isolated studies may not adequately depict the complexities of real-world exposure settings in which individuals are exposed to many PFAS and metals at the same time [22,23]. People are exposed to PFAS and metal combinations through various sources, including polluted drinking water, food, and consumer products [24,25]. As a result, examining cumulative exposure to multiple PFAS and metals rather than individual ones provides a more realistic picture of the environmental and health concerns associated with PFAS and metals [26,27]. 

#### 1.1.5. Allostatic Load

Allostatic load is “the wear and tear on the body” that occurs when an individual is subjected to recurrent or chronic stress. It represents the physiological implications of recurrent or sustained chronic stress exposure to fluctuating or heightened neuronal or neuroendocrine response [28,29]. Allostatic load is an emerging concept in hepatic disease research, as chronic stress has been shown to contribute to liver dysfunction and disease progression [30]. Understanding the relationship between combined PFAS exposure, metals exposure, and allostatic load can provide insights into the mechanisms underlying the association between PFAS exposure, metals exposure, and hepatic diseases.

### 1.2. Research Objectives

The major purpose of this study is to investigate the relationship between combined PFAS/metals exposure and allostatic load on hepatic disease risk. This study seeks to shed light on a potentially essential yet understudied mechanism linking environmental factors to liver health by exploring the interaction between PFAS/metals.

This study is significant as it can inform public health interventions and regulatory measures to reduce PFAS and metals exposure and prevent hepatic diseases. By identifying the association between combined PFAS exposure and hepatic disease risk, policymakers and healthcare professionals can develop strategies to mitigate the adverse health effects of PFAS exposure.

## 2. Materials and Methods

### 2.1. Study Design

For this study, the data source is the National Health and Nutrition Examination Survey (NHANES) data from 2017–2018 among a representative sample of the U.S. non-institutionalized civilian population, involving 9254 participants. NHANES is a program conducted by the Centers for Disease Control and Prevention (CDC) to assess the health and nutritional status of adults and children in the United States. NHANES utilizes a complex, multistage, stratified sampling design to represent non-institutionalized civilians in the United States [31,32].

The NHANES survey covers questions regarding demographics, socioeconomic status, diet, and health, followed by medical, dental, and physiological tests and laboratory examinations conducted by trained medical professionals. Its findings are vital for determining disease prevalence, assessing nutritional status, setting national standards for health measurements, and supporting public health policy and programs [31,32].

#### Description of Cohort

The NHANES provided data for the study. Between 2017 and 2018, the National Center for Health Statistics (NCHS), a part of the Centers for Disease Control and Prevention (CDC) in the United States, gathered the data [31,32]. Leveraging NHANES data, our study sought to determine whether exposure to PFAS and metals in combination affected the liver. A variety of PFAS, including PFOS and PFOA, and metals such as Pb, Cd, and Hg were investigated in this study. The study also looked at the allostatic load index using a range of clinical and bio-markers, such as albumin, creatinine clearance, BMI, triglycerides, HDL cholesterol, total cholesterol, CRP, and hemoglobin A1C [33].

### 2.2. Blood Measurement Sample

For the NHANES, a mobile exam center (MEC) gathered blood samples for laboratory processing, including evaluations for exposure to environmental agents such as PFAS and metals. Under carefully monitored circumstances, these samples were prepared, kept, and sent to laboratories around the nation [33].

### 2.3. Operationalizing Allostatic Load

Drawing from prior research, allostatic load was measured by aggregating dysfunction across various physiological systems, including the cardiovascular system (systolic blood pressure (SBP), diastolic blood pressure (DBP), triglycerides, high-density lipoprotein (HDL) cholesterol, total cholesterol, the metabolic system (albumin, body mass index (BMI), hemoglobin A1C, and creatinine clearance) and the inflammatory system, c-reactive protein (CRP) [5,34,35,36]. These markers were categorized into quartiles based on their distribution within the database. The high-risk category was identified as the top quarter in the distribution for most markers, except for albumin, creatinine clearance, and HDL cholesterol, for which the highest risk was the lowest quarter of the distribution. Each study participant received a value of “0” if categorized in the lower-risk group and “1” if placed in the high-risk group, resulting in a total allostatic load score out of 10 [16].

### 2.4. Utilization of United States-Fatty Liver Index for Non-Alcoholic Fatty Liver Disease Prediction

Non-alcoholic fatty liver disease is a prevalent chronic liver condition characterized by the accumulation of fat in the liver in individuals who do not consume excessive alcohol. It has become a significant public health concern globally, particularly in the United States, where its prevalence is on the rise. Accurate and reliable methods for early detection and prediction of non-alcoholic fatty liver disease are crucial for effective management and intervention strategies.

In the absence of abdominal ultrasound data within the dataset utilized, the United States-fatty liver index (US-FLI) was employed as an alternative method for predicting non-alcoholic fatty liver disease. The reliability of the US-FLI as an indicator for non-alcoholic fatty liver disease within the United States population has been established in prior research. The computation of the US-FLI is based on a logistic regression formula that estimates the likelihood of non-alcoholic fatty liver disease occurrence based on variables such as body mass index (BMI), waist measurement, gamma-glutamyl transferase (GGT), and triglycerides (TG), detailed in the equation below:

$$FLI=\frac{exp(A)}{1+exp(A)}\times 100,$$

where the variable A is defined as:

A = 0.953 × log(TG) + 0.139 × BMI + 0.718 × log(GGT) + 0.053 × waist circumference − 15.745.

### 2.5. Hypothesis

In this study, we hypothesized that there is a significant association between the combined exposure to PFOA, PFOS, metals, and allostatic load and an increased risk of hepatic disease. We aim to examine whether individuals exposed to these chemicals and stress-related biological markers are at a higher risk of developing liver-related health. This hypothesis is based on the premise that the interaction between these environmental and physiological factors could contribute to the onset or progression of hepatic disease.

### 2.6. Statistical Analysis

The statistical analysis within the study by our team commenced with the application of descriptive statistics, which facilitated the examination of mean values, standard errors, and confidence intervals for demographic variables and various environmental contaminants such as Cd, Pb, Hg, PFOA, and PFOS. 

Subsequently, linear regression analysis was conducted to assess the impact of these environmental contaminants on AST, ALT, ALP, and total bilirubin levels, with adjustments made for age and alcohol consumption. 

Further analysis employed Bayesian kernel machine regression (BKMR) [37,38] to investigate the complex and potentially synergistic relationships between these contaminants and the US-FLI, a measure of the likelihood of non-alcoholic fatty liver disease disease. This advanced statistical approach adjusted for a set of covariates, including BMI, sex, age, ethnicity, alcohol consumption, and smoking, to refine the understanding of the contaminants’ impact on liver health. The software R (version 4.2.3 R Foundation for Statistical Computing, Vienna, Austria) was utilized to perform the BKMR and generate Spearman plots, while Stata SE 18 (StataCorp, College Station, TX, USA) was applied for conducting descriptive statistics and linear regression analysis. Significance throughout the study was determined at the *p* < 0.05 level. Subsample weights and adjustments for the complex survey design were incorporated into all analyses except for BKMR, which does not support these adjustments.

In this study, we employed distinct sets of confounders for linear regression and Bayesian kernel machine regression (BKMR) to align with the methodological strengths of each modeling technique. BKMR is adept at managing numerous variables simultaneously and can capture complex interactions and non-linear relationships. Therefore, in the BKMR analysis, we included a comprehensive set of confounders, namely body mass index (BMI), sex, age, ethnicity, alcohol consumption, and smoking status, to fully leverage its analytical capabilities.

Conversely, linear regression, with its more restrictive assumptions and limited capacity to handle multicollinearity and complex interactions, necessitated a more selective approach to confounder inclusion. Thus, for the linear regression model, we focused on the most critical variables based on their established impact on the outcome, adjusting for age and alcohol consumption only. This approach ensured that the linear regression model remained robust and statistically sound while acknowledging its inherent limitations in handling many predictor variables.

## 3. Results

### 3.1. Descriptive Statistics of Key Study Variables

Table 1 shows the mean values for the variables of interest in the study across male and female participants, highlighting mean values, standard errors (SE), and 95% confidence intervals (CI) for age and several environmental contaminants. These findings suggest significant sex differences in the levels of certain environmental contaminants. Particularly, males exhibited higher levels of PFOA, PFOS, and Pb, whereas females had higher levels of Cd. The almost equal levels of mercury across sexes suggest a uniform exposure or bioaccumulation pattern for this particular contaminant. Regarding smoking, among those who smoked every day, males represented 29.3 percent, and females represented 37.5 percent. For those who smoked some days, males represented 8.34 percent and females 9.57 percent. Among those who did not smoke, males represented 62.3 percent and females 52.9 percent. The ethnic makeup of the study was 10.8 percent Mexican American, 7.27 percent Other Hispanic, 59.13 percent Non-Hispanic White, 11.84 percent Non-Hispanic Black, 5.59 percent Non-Hispanic Asian, and 5.32 percent Other-Race Including Multi-Racial. These results provide valuable insights into the sex-specific differences in environmental exposure.

### 3.2. Linear Regression of Environmental Exposures plus Allostatic Load on Liver Injury Markers

We analyzed the data using linear regression to understand the association of PFOA, PFOS, Pb, Hg, Cd, and allostatic load on individual liver injury markers. Firstly, we performed linear regression to assess the effects of environmental exposure and allostatic load on AST, as shown in Table 2. The regression model revealed that PFOA, Pb, and Cd are significant with a *p*-value less than 0.05 level of significance. Meanwhile, PFOS, Hg, and allostatic load were not significantly associated with AST. 

The association between the exposure variables and allostatic load with ALT levels was explored. The results can be found in Table 3. The regression model reveals that Pb and Cd are significantly associated with AST with *p*-values less than 0.05 level of significance, while PFOA, PFOS, Hg, and allostatic load were not significant in the model predicting ALT. 

Table 4 shows the association between exposure variables and ALP. The results reveal a significant association of ALP with mercury. 

Table 5 shows the association between exposure variables and total bilirubin. The results reveal significant associations of total bilirubin with Pb, Cd, and allostatic load. 

### 3.3. Spearman Correlational Analysis between Environmental Exposures and Liver Injury

**Figure 1:**
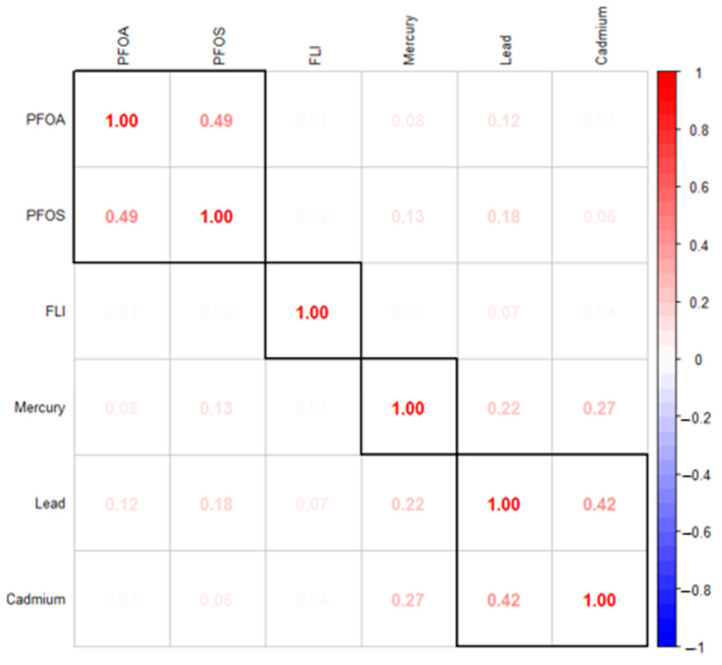
Spearman correlation analysis of critical study variables.

To explore the relationship between variables, we performed a correlational analysis. Figure 1 presents the Spearman correlation analysis conducted using PFOA, PFOS, Hg, Cd, Pb, and the fatty liver index (FLI), a measure of the likelihood of non-alcoholic fatty liver disease. The results indicate moderate correlations within the group of metals and within the group of PFAS, respectively, as opposed to the correlations between metals and PFAS. 

### 3.4. Bayesian Kernel Machine Regression Analysis of Environmental Exposures with the Fatty Liver Index

#### 3.4.1. Posterior Inclusion Probabilities (PIP)

We explored the role of PFAS and metals on the likelihood of non-alcoholic fatty liver disease using Bayesian kernel machine regression analysis. This advanced statistical approach allowed us to unravel the complex, potentially synergistic relationships between multiple environmental contaminants and liver health. The analysis commenced with exploring the posterior inclusion probabilities (PIP), a critical step for identifying the contaminants that contribute most to the likelihood of non-alcoholic fatty liver disease. The PIP values serve as a robust indicator of the likelihood that each specific contaminant plays a significant role in the model predicting the likelihood of non-alcoholic fatty liver disease. A high PIP value for a contaminant suggests strong evidence of its association with alterations in the fatty liver index, indicating its importance in the multi-contaminant exposure framework.

Our analysis, as presented in Table 6, revealed that Pb, Hg, and PFOS are the contaminants with the highest PIP values. This finding implies that among the array of PFAS and metals analyzed, these three substances have the most substantial evidence pointing to their influential roles in affecting liver health, as indicated by their associations with the fatty liver index. 

#### 3.4.2. Univariate Analysis: Examining the Isolated Effects of PFOA, PFOS Hg, Cd, and Pb on FLI

**Figure 2:**
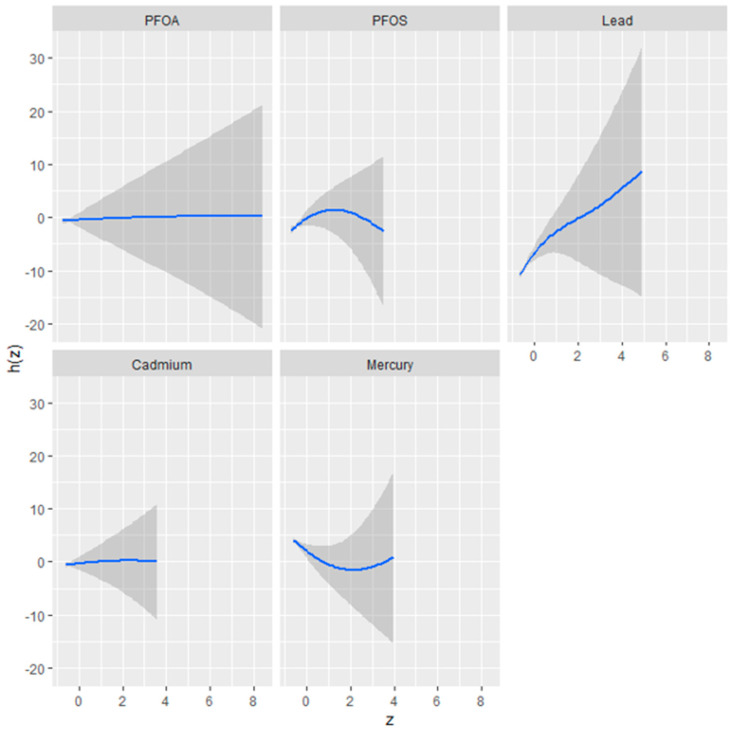
Univariate exposure–response functions and 95% confidence interval (dark grey color) for the association between single metal/PFAS exposure when other metals/PFAS exposures are fixed at the median.

Figure 2 presents the results of a univariate analysis exploring the isolated effects of five environmental contaminants—perfluorooctanoic acid (PFOA), perfluorooctanesulfonic acid (PFOS), mercury (Hg), cadmium (Cd), and lead (Pb)—on the fatty liver index, a predictor of liver health. The figure comprises a series of plots, each corresponding to one of the contaminants, illustrating the relationship between the contaminant concentration (as indicated by the *z*-axis) and the fatty liver index (h[Z]).

The plots reveal distinct exposure–response relationships, characterized by the shape and spread of the shaded areas, which represent the 95% confidence intervals. A steep slope or curve within a plot suggests a stronger association between the contaminant concentration and changes in the fatty liver index.

The plot shows a relatively flat response for PFOA, indicating a minimal or non-linear effect on the fatty liver index. PFOS, conversely, may exhibit a non-linear relationship, where the impact on the fatty liver index initially changes with concentration but plateaus or changes direction beyond a certain threshold.

Pb appears to demonstrate a more pronounced positive relationship with the fatty liver index, as suggested by a plot with a positive slope, implying that higher concentrations of Pb are associated with an increase in the fatty liver index.

Cd might display a unique response pattern, perhaps an inverted U-shape, signifying a complex relationship where the effect on the fatty liver index increases up to a certain concentration level before declining.

Hg’s plot could present a U-shaped or non-linear relationship, indicating that changes in mercury levels are associated with fluctuations in the fatty liver index, which could signify varying impacts at different concentration levels.

Understanding these isolated effects is crucial for discerning the potential risks associated with each substance and informs subsequent multivariate analyses that consider the combined impact of these environmental factors.

### 3.5. Visualizing Bivariate Exposure–Response Functions with Fixed Percentile Values

**Figure 3:**
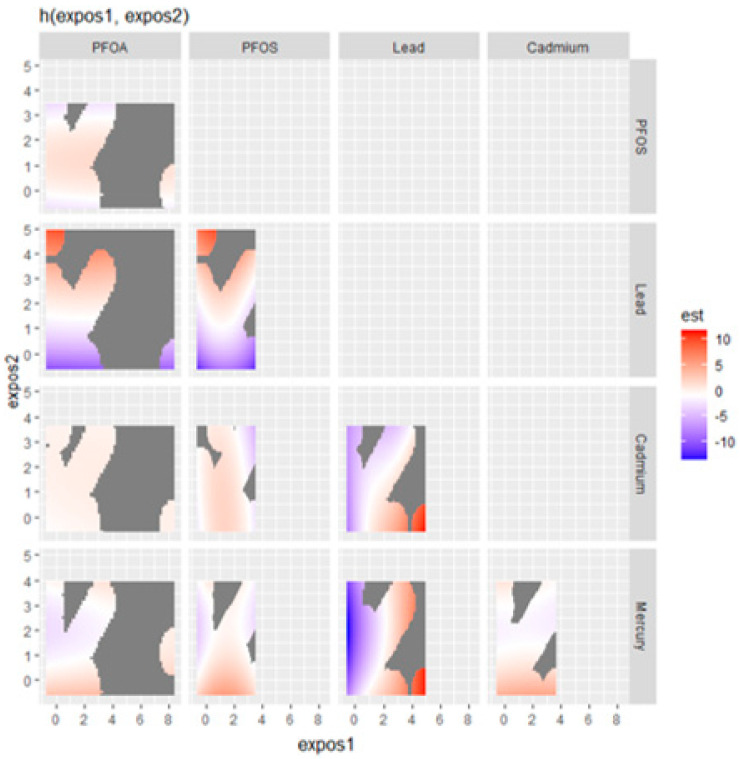
Bivariate exposure–response function of metals with CRP.

In the exploration of the impact of combined metal and PFAS exposure on the fatty liver index, Figure 3 presents a nuanced visualization of how pairs of contaminants—specifically Hg, Cd, Pb, PFOS, and PFOA—interact to affect liver health. This analysis fixes all other predictors at a certain percentile to isolate the effects of the contaminants of interest.

The plot indicates a potentially synergistic effect between Hg and Cd, where their concurrent high levels are associated with an elevated fatty liver index, as denoted by the red area. Conversely, the interaction of Hg with Pb suggests that increased Pb levels could drive up the fatty liver index, particularly when Hg levels are not at their highest. This relationship is visualized through a gradient transitioning from orange to red.

The interplay between Hg and PFOS appears more complex; at lower PFOS concentrations, an increase in Hg correlates with a higher fatty liver index, a relationship that seems to wane or even invert at higher PFOS levels. In contrast, Cd and Pb together demonstrate a clear pattern where their combined high levels correlate with an increased fatty liver index, hinting at a more pronounced joint impact on liver health.

Interestingly, the Cd and PFOS plot suggests an antagonistic interaction, where the presence of high PFOS levels may attenuate the impact of Cd on the fatty liver index, observable through a color shift from red to blue. Similarly, PFOS, combined with high levels of Pb, seems to amplify the fatty liver index, as indicated by the prominent red regions for these higher concentration combinations.

However, the relationship between Pb and PFOA doesn’t manifest a clear pattern, suggesting that their combination may not consistently influence the fatty liver index. The plot examining PFOA and PFOS interactions indicates a non-linear relationship, with moderate levels of both contaminants associated with higher fatty liver index values but without a proportional increase at the highest contaminant levels.

Moreover, the interaction between Hg and PFOA does not seem to exhibit a strong association with the fatty liver index, as no significant color gradients suggest such a link. Similarly, for cadmium and PFOA, the evidence points to only a modest interaction effect on the fatty liver index at certain exposure levels, lacking a pronounced pattern.

These visual findings elucidate the complex and varied interactions between specific metals and PFAS, offering critical insights into their potential combined effects on liver health, which could be instrumental for health risk assessment and regulatory policies.

**Figure 4:**
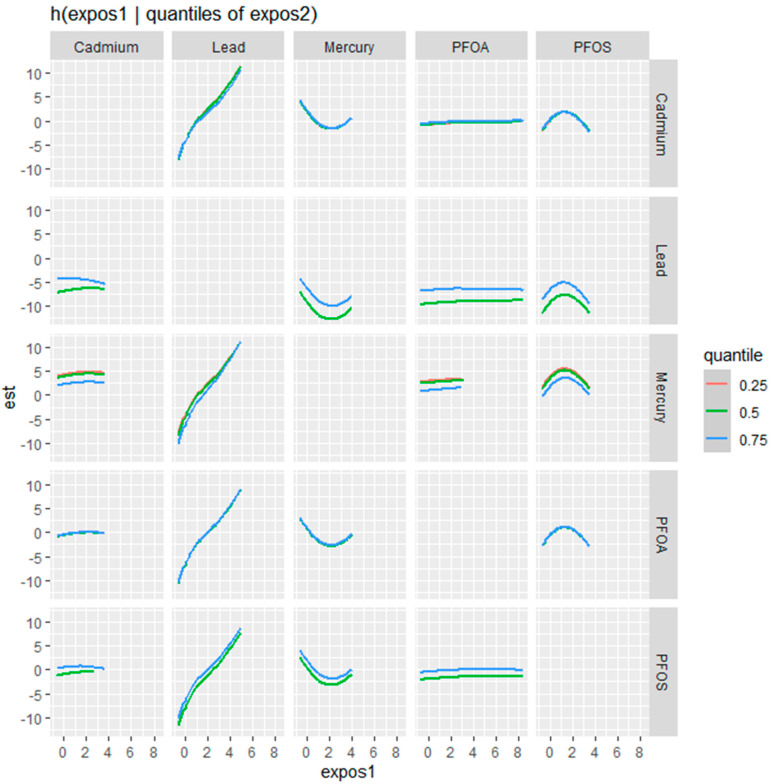
Bivariate exposure–response function of metals with CRP—investigating predictor–response function with varying quantiles of the second predictor, while other predictors are fixed.

Figure 4 examines the bivariate relationships between Cd, Pb, Hg, PFOA, and PFOS with the fatty liver index, showcasing a nuanced interplay where the influence of one contaminant is modulated by the concentration of another. This modulation is assessed at different quantile levels—25th (blue line), 50th (green line), and 75th (red line)—of the second contaminant, offering a stratified perspective on the risk each contaminant poses to liver health at varying exposure levels.

For Cd, the effect on the fatty liver index becomes more pronounced with increasing levels of the second contaminant, as reflected by the steepening slope from the blue to the red line. This suggests that higher concentrations of other contaminants may amplify Cd’s impact. In the case of Pb, a similar pattern emerges, with a notable increase in the fatty liver index, especially at the higher quantiles of co-exposure, indicated by the sharper ascent of the red line.

Hg’s relationship with the fatty liver index is characterized by a U-shaped curve at the median quantile of the second contaminant, with the effect being less defined at the lower and higher quantiles. This pattern suggests that the impact of mercury on the fatty liver index is most distinct when other contaminants are at their median levels.

Conversely, PFOA demonstrates a relatively stable profile, with the effect on the fatty liver index showing little variation across the different quantiles of the second contaminant, indicating potential independence from the influence of co-exposures.

PFOS displays a more variable relationship, with its impact on the fatty liver index intensifying at the median to higher levels of the second contaminant. This is particularly evident from the green and red lines, where the increase in the fatty liver index is more pronounced.

### 3.6. Overall Risk Summary of Fatty Liver Index Levels in Relation to Exposure Percentiles

**Figure 5:**
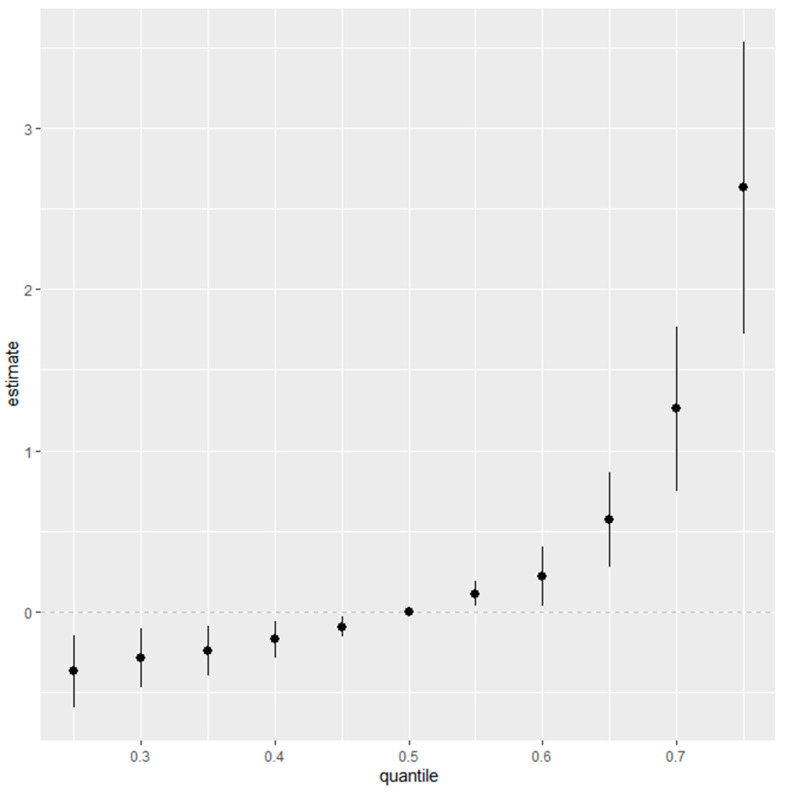
Summary of overall health effects of the exposures (multimixers) on the outcome depends on various percentiles (from 25th to 75th percentiles).

Figure 5 represents an aggregated risk assessment (overall risk summary) for the fatty liver index, taking into account the entire spectrum of environmental exposures, specifically Cd, Pb, Hg, PFOA, and PFOS. The analysis demonstrates the collective effect of these contaminants, fixed at different quantiles ranging from the 25th to the 75th percentile, with increments of 5 percentiles, and uses the median, or the 50th percentile, as a benchmark for comparison.

Initially, at the 25th percentile, representing the lower end of exposure, the risk posed by the combination of Cd, Pb, Hg, PFOA, and PFOS to the fatty liver index is minimal, as indicated by the estimates clustering near zero. This observation implies that lower levels of these contaminants may not have a significant impact on the fatty liver index. Moving through the exposure distribution, the estimates appear stable as they approach and include the 50th percentile, suggesting a uniform effect of median exposure levels on the fatty liver index.

However, the trend shifts notably above the median percentile. Particularly beyond the 50th percentile, the estimate of the combined effect escalates, reaching its apex at the 75th percentile. The widening of the confidence intervals accompanying this uptrend suggests growing uncertainty, possibly reflective of individual variabilities in response to higher contaminant levels or due to the diminishing number of observations at these upper exposure ranges.

The most pronounced effect is at the 75th percentile, where the plot shows a significant increase in the estimate’s magnitude. This inflection point underscores a heightened association between higher exposure levels to Cd, Pb, Hg, PFOA, and PFOS, and an elevated fatty liver index. The implication is clear: individuals who fall into the higher percentile of exposure to these specific contaminants carry a greater risk for conditions associated with fatty liver disease.

In weaving together these observations, the figure highlights that the risk to liver health from environmental exposures is not equally distributed across exposure levels. Instead, it underscores the escalating nature of risk associated with the higher cumulative exposure to Cd, Pb, Hg, PFOA, and PFOS. This insight is critical for public health strategies, emphasizing the need to mitigate exposure, particularly for those in the higher percentile brackets, to reduce the potential for liver-related health outcomes.

### 3.7. Single-Variable Effects of Metals and PFAS on the Fatty Liver Index

**Figure 6:**
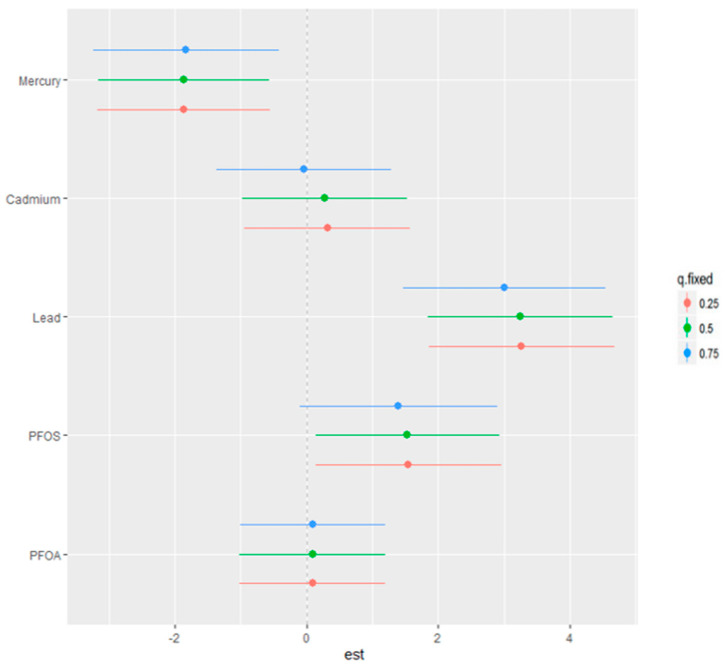
Single-variable effect of metals and PFAS at increasing quartiles for the fatty liver index.

Figure 6 provides an insight into the single-variable effects of various environmental contaminants on the fatty liver index, examining the influence of each predictor individually at different levels of exposure—represented by the 25th (red), 50th (green), and 75th (blue) percentiles. This detailed view allows us to dissect their individual contributions to the risk of an elevated fatty liver index, reflecting the isolated impact of Hg, Cd, Pb, PFOS, and PFOA.

The figure’s horizontal lines represent the confidence intervals for the estimates at each percentile, with the colored points marking the central estimate. The h function, a flexible statistical construct, considers the multiple metals and PFAS, combining them to model the complex and potentially non-linear relationship between these exposures and the fatty liver index.

Overall, Pb and PFOS have the most profound impact on the fatty liver index at all exposure levels.

From top to bottom, starting with Hg, it appears that the central estimates suggest an increase in the fatty liver index at the 75th percentile, denoted by the red point, indicating a higher contribution to the overall risk at higher exposure levels. For Cd, the central estimates across the three quantiles are relatively close to or below zero, suggesting a less pronounced or potentially negligible single-variable effect on the fatty liver index, with exposure at the 75th percentile having the largest impact on the fatty liver index.

Moving to Pb, the estimates again rise notably at the 75th percentile, implying that elevated levels of Pb are associated with higher values of the h function and, thereby, a greater risk to liver health. PFOS and PFOA both demonstrate central estimates that increase across the exposure quantiles. Particularly for PFOS, the increase in the estimate from the 25th to the 75th percentile is evident, with the highest percentile showing a notably higher effect size.

The interpretation of these results points to a gradation in the single-variable effect of these contaminants on the fatty liver index, with Hg, Pb, and PFOS suggesting a more substantial influence, especially at higher exposure levels. This quantile-based approach underscores the importance of considering how different levels of exposure can variably impact the risk of liver conditions, emphasizing the need for targeted risk assessments and interventions for individuals with higher exposure to these specific contaminants.

### 3.8. Differential Risk Assessment of Single Contaminant Exposure on the Fatty Liver Index

**Figure 7:**
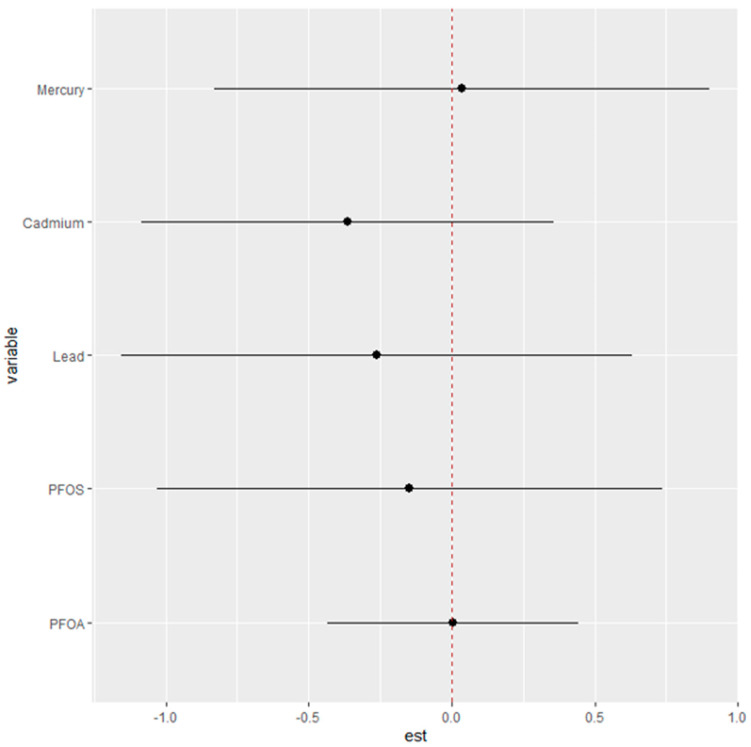
Single-exposure risk estimates for mercury, cadmium, lead, PFOS, and PFOA on the fatty liver index, comparing the change in risk associated with each contaminant from its 25th to 75th percentile. The plot illustrates the differential risk impact when other exposures are fixed at their 25th percentile (left end of the horizontal line) versus their 75th percentile (right end of the horizontal line), with the point estimates indicating the magnitude of change and the horizontal lines representing the confidence intervals.

Figure 7 seeks to elucidate the specific “interaction” parameters between different environmental exposures and their associated health risks. The objective is to discern how the risk attributed to a single exposure changes when the context of other exposures is altered.

In this analysis, the health risks of single exposures—Hg, Cd, Pb, PFOS, and PFOA—are evaluated under two contrasting scenarios: first, when all other exposures are fixed at their 25th percentile, and second, when they are set at their 75th percentile. The plot provides estimates (est) that reflect the magnitude of change in risk associated with a single exposure as it moves from its 25th to 75th percentile within these two different contextual backdrops of other exposures.

The dots represent the estimated change in risk for each exposure, while the horizontal lines denote the confidence intervals around these estimates. If the dot is positioned to the right of the zero line, it suggests an increase in the single-exposure risk associated with the transition from its 25th to 75th percentile. Conversely, if the dot is to the left, it indicates a decrease in risk.

From the plot, we can infer that the single-exposure risk for each metal or PFAS changes by certain units when the remaining exposures are fixed at their 25th percentile compared to when they are fixed at their 75th percentile.

## 4. Discussion

The findings from the study provide an assessment of the impact of environmental contaminants on liver health, specifically focusing on the role of Pb, Cd, Hg, PFOA, and PFOS on AST, ALT, GGT, ALP, total bilirubin, and the fatty liver index.

The linear regression analysis conducted in the study aimed to determine the effects of a few environmental contaminants on liver injury enzymes. The regression model identified PFOA, Pb, and Cd as significant predictors of AST levels, with *p*-values less than 0.05, indicating a statistically significant association with AST levels. For ALT levels, significant associations were found between Pb and Cd. For ALP, significant associations were found with Hg. Significant associations were found with Pb, Cd, and allostatic load for total bilirubin. The results indicate that metals have a profound effect on the hepatic system. This matches the work of others who have made similar findings [39]. The relationship between total bilirubin and allostatic load suggests that the processes influencing bilirubin levels might reflect broader systemic stress responses, not just localized liver health issues [40,41]. Simply put, the association of bilirubin with allostatic load could imply that external social stressors not only affect mental health but may also have tangible physiological manifestations that can be tracked through biomarkers like bilirubin.

Spearman correlation analysis underscored positive correlations among the metals and a positive correlation between PFAS, indicating interrelated exposure patterns among these environmental factors. The correlations suggest that these contaminants may have common sources or release mechanisms in the environment [42]. For example, industrial processes, waste disposal practices, or consumer products could be releasing both metals and PFAS, leading to simultaneous exposure in nearby populations. Understanding that these contaminants may come from similar sources can inform more effective regulatory and remediation strategies. Targeting shared sources could simultaneously reduce exposure to multiple hazardous substances [43]. Overall, the interrelated exposure patterns indicate that individuals exposed to one type of contaminant (e.g., metals) are likely to be exposed to others (e.g., PFAS), potentially compounding the health risks associated with each contaminant.

Through BKMR analysis, we investigated the multifaceted and potentially synergistic influences of PFAS and metals on the likelihood of non-alcoholic fatty liver disease as measured by the fatty liver index. This advanced statistical approach was pivotal in disentangling the complex relationships between a multitude of environmental contaminants and liver health indicators. A key step in our analysis was examining posterior inclusion probabilities (PIP), which serve as a robust measure of a contaminant’s likelihood to significantly impact the model predicting the likelihood of non-alcoholic fatty liver disease. High PIP values indicate strong associations with alterations in the fatty liver index and denote the importance of the contaminant within the multi-contaminant exposure framework.

Pb, Hg, and PFOS emerged with the highest PIP values, signaling their significant roles in affecting liver health. Notably, lead showed a strong positive relationship with the fatty liver index, suggesting that higher concentrations may contribute to an elevated likelihood of non-alcoholic fatty liver disease. Hg and Cd displayed complex, non-linear relationships with the fatty liver index, characterized by U-shaped or inverted U-shaped response patterns, indicating variable impacts at different concentration levels. The results for Pb, Hg, and PFOS suggest that remediation strategies in the context of the exposome may require focusing on these chemicals [44].

These results carry substantial implications for public health, highlighting the need for targeted interventions to reduce exposure to these contaminants [45]. The associations found between these metals and PFAS with the fatty liver index suggest that even at the individual exposure level, there is a discernible risk for liver health compounded by other environmental contaminants.

The univariate BKMR analysis, showcasing the isolated effects of PFOA, PFOS, Hg, Cd, and lead on the fatty liver index, found Pb exhibiting a strong positive slope, implying a dose-dependent increase in the likelihood of non-alcoholic fatty liver disease. Conversely, Cd and Hg showed non-linear relationships, suggesting complex dynamics at varying concentrations. Such detailed univariate insights are integral to understanding the potential risks of each substance before considering their combined effects [37].

The bivariate exposure–response functions suggested potential synergistic effects among certain contaminants. For instance, when Hg and Cd are both present at high levels, there seems to be a compounded increase in the likelihood of non-alcoholic fatty liver disease. This could imply a synergistic effect that exacerbates liver health risk when both contaminants are elevated [46]. Conversely, the combination of Hg and Pb presents a gradient of effect, with higher levels of Pb associated with an increased likelihood of non-alcoholic fatty liver disease, especially when Hg is not at its peak levels. This speaks to the complexity of exposure and the need to plan for multiple exposure concentrations and combinations to best capture the effects of multiple exposures on disease risk [47].

Moreover, the interaction between Hg and PFOS revealed a complex relationship where low PFOS concentrations coupled with rising Hg levels correlated with a higher likelihood of non-alcoholic fatty liver disease, a relationship that seemed to diminish or reverse at higher PFOS concentrations. This finding suggests a non-linear interaction where the effects of one contaminant may be modulated by the presence of another. Interestingly, Cd and PFOS interactions suggest an antagonistic effect, where high PFOS levels might mitigate cadmium’s impact on FLI. Such antagonistic interactions could be crucial in understanding the multifaceted nature of contaminant effects on liver health [48].

Additional exploration of the bivariate relationships examining the influence of one contaminant while varying the levels of a second contaminant across the 25th, 50th, and 75th percentiles provided profound results. This analysis revealed that Cd’s impact on the likelihood of non-alcoholic fatty liver disease is more pronounced with increasing levels of a co-occurring contaminant, while Pb showed a sharper increase in the likelihood of non-alcoholic fatty liver disease at higher quantiles of co-exposure. Mercury’s impact on the likelihood of non-alcoholic fatty liver disease was most distinct at median levels of other contaminants, suggesting that its effect is not linear but rather influenced by other substances’ presence. PFOS shows a variable relationship, with its impact on the likelihood of non-alcoholic fatty liver disease intensifying at the median to higher levels of co-exposure. These findings speak to the complexity and multifaceted criteria that must be considered when exploring exposure to multiple contaminants. Exposure is complex, and when also considered in the context of health, the dynamics become even more profoundly complex [49].

The single-variable effects of these contaminants on non-alcoholic fatty liver disease were also explored. Here, the analysis reflects isolated impacts at varying exposure levels, providing insight into how each contaminant individually contributes to the risk of an elevated likelihood of non-alcoholic fatty liver disease. Notably, Hg, Pb, and PFOS exhibited a more substantial influence on the risk, particularly at higher exposure levels. This gradation, in effect, as reflected by different quantiles, indicates that risk to liver health is not uniform across exposure levels and highlights the need for public health interventions that are tailored to individual exposure profiles [50].

A more granular assessment of single-variable effects at different exposure quantiles (25th, 50th, and 75th) was also explored. These figures laid out a quantile-based gradation of risk, with Hg, Pb, and PFOS showing more pronounced effects at higher exposure levels. For instance, at the 75th percentile, the central estimates for Hg and Pb indicated a higher contribution to overall risk, while PFOS showed a notable increase in effect size from the 25th to the 75th percentile. This approach highlighted the importance of targeted risk assessments and interventions for those with higher exposure levels [51].

Finally, analysis examining the “interaction” parameters by comparing the risk changes for a single exposure when other exposures were fixed at their lower (25th percentile) versus higher (75th percentile) levels offered critical insights. This differential assessment revealed how single-exposure risks, such as those from Hg or Pb, varied depending on the background levels of other contaminants, providing critical insights for risk mitigation strategies. Collectively, these findings from the BKMR analysis have significant implications for public health, particularly in the realm of environmental exposure and liver disease prevention. They underscore the need for policies and interventions that address individual contaminant risks and the combined exposure scenarios that may elevate health risks.

### Limitations

This study is not without limitations. Firstly, the cross-sectional study design, which involves data collection at a single point in time, restricts the researchers’ ability to establish a cause-and-effect relationship or infer the temporal sequence of events. Consequently, it becomes challenging to determine whether the observed environmental factors preceded or resulted from the liver injury outcomes. Furthermore, while the study provides valuable insights into the collective impact of environmental factors on liver injury, it falls short of establishing causality. Despite these limitations, the research contributes valuable information that enhances our understanding of how various environmental factors may collectively influence liver health, offering a foundation for further investigations and potentially guiding public health interventions.

## 5. Conclusions

This study, utilizing NHANES 2017–2018 data, provides insights into the impact of environmental contaminants—specifically PFOA, PFOS, metals, and allostatic load—on liver health markers, including AST, ALT, ALP, total bilirubin, and the fatty liver index. Descriptive statistics, Spearman’s correlation analysis, linear regression, and BKMR results reveal significant associations and complex interactions among these exposures, highlighting their collective impact on liver function. The findings underscore the necessity for public health strategies that address the multifaceted nature of environmental exposures to mitigate risks of hepatic diseases. Future research should focus on longitudinal studies to further elucidate these relationships and inform more targeted interventions. As suggested by the study, targeted interventions would be specifically designed to address the nuanced impacts of environmental contaminants like PFOA, PFOS, and metals on liver health. These interventions could focus on high-risk communities or populations, offering more precise and effective strategies tailored to the identified unique exposure profiles and health needs. By homing in on the specific sources and types of environmental exposure linked to liver disease, public health officials can craft more efficient and direct measures to mitigate risk.

## Figures and Tables

**Table 1:** Mean values for variables of interest.

	Male (Mean)	95% CI (Male)	SE (Male)	Female (Mean)	95% CI (Female)	SE (Female)
Age *n* = 9254	37.42	(36.37, 38.47)	0.493	39.37	(38.12, 40.63)	0.592
BMI *n* = 8005	27.515	(26.97, 28.06)	0.257	27.932	(27.27, 28.60)	0.313
AL *n* = 3233	3.442	(3.284, 3.600)	0.074	3.591	(3.456, 3.726)	0.063
PFOA *n* = 2479	1.874	(1.717, 2.032)	0074	1.604	(1.423, 1.785)	0.084
PFOS *n* = 2479	6.817	(6.108, 7.527)	0.333	4.780	(4.213, 5.347)	0.265
LEAD *n* = 7434	1.162	(1.084, 1.240)	0.036	0.869	(0.824, 0.913)	0.021
Cadmium *n* = 8063	0.326	(0.306, 0.346)	0.009	0.426	(0.388, 0.464)	0.018
Mercury *n* = 8063	1.189	(1.031, 1.347)	0.074	1.092	(0.976, 1.208)	0.054

SE *=* standard error. CI *=* confidence interval.

**Table 2:** Association between exposure variables and allostatic load with AST.

AST *	Coefficient	95% CI	SE	*p*-Value
PFOA	0.923	(0.109, 1.738)	0.382	0.029
PFOS	−0.081	(−0.205, 0.043)	0.059	0.183
Lead	0.0539	(0.361, 0.716)	0.083	<0.0001
Cadmium	−0.972	(−1.895, 0.048)	0.433	0.040
Mercury	−0.134	(−0.875, 0.607)	0.347	0.705
Allostatic load	−0.485	(−1.410, 0.437)	0.433	0.279

* Adjusted for age and alcohol consumption. SE *=* standard error; CI *=* confidence interval.

**Table 3:** Association between exposure variables and ALT.

ALT *	Coefficient	95% CI	SE	*p*-Value
PFOA	1.030	(−0.171, 2.230)	0.563	0.087
PFOS	−0.036	(−0.224, 0.152)	0.088	0.686
LEAD	1.087	(0.862, 1.311)	0.105	<0.0001
Cadmium	−3.092	(−4.641, −1.543)	0.727	0.001
Mercury	−0.129	(−1.069, 0.810)	0.441	0.773
Allostatic load	0.269	(−0.955, 1.492)	0.574	0.647

* Adjusted for age and alcohol consumption. SE *=* standard error; CI *=* confidence interval.

**Table 4:** Association between exposure variable and ALP.

ALP *	Coefficient	95% CI	SE	*p*-Value
PFOA	−0.0585	(−2.356, 2.239)	1.078	0.957
PFOS	0.0468	(−0.174, 0.267)	0.103	0.657
Lead	−0.1198	(−0.545, 0.306)	0.200	0.557
Cadmium	2.188	(−2.588, 6.963)	2.241	0.344
Mercury	−2.739	(−4.146, −1.332)	0.660	0.001
Allostatic load	1.263	(−0.691, 3.217)	0.917	0.188

* Adjusted for age and alcohol consumption. SE = standard error; CI = confidence interval.

**Table 5:** Association between exposure variables and total bilirubin.

Total Bilirubin *	Coefficient	95% CI	SE	*p*-Value
PFOA	0.022	(−0.005, 0.048)	0.012	0.102
PFOS	0.004	(−0.000, 0.008)	0.002	0.064
LEAD	0.010	(0.005, 0.015)	0.002	0.001
Cadmium	−0.077	(−0.104, 0.050)	0.013	<0.0001
Mercury	−0.001	(−0.024, 0.023)	0.011	0.943
Allostatic load	−0.024	(−0.036, 0.013)	0.006	<0.001

* Adjusted for age and alcohol consumption. SE = standard error; CI = confidence interval.

**Table 6:** Posterior inclusion probabilities for the metals and PFAS on the likelihood of non-alcoholic fatty liver disease.

Variable	PIP
PFOA	0.1926
PFOS	0.5842
Lead	1.000
Cadmium	0.3418
Mercury	0.7470

## Data Availability

The NHANES dataset is publicly available online, accessible at https://www.cdc.gov/nchs/nhanes/index.htm (accessed on 12 December 2023).
